# Effectiveness of Combined Oral Minoxidil and Finasteride in Male Androgenetic Alopecia: A Retrospective Service Evaluation

**DOI:** 10.7759/cureus.77549

**Published:** 2025-01-16

**Authors:** Hans Johnson, David Huang, Ashley K Clift, Ângela Bersch-Ferreira, Gabriel A Guimarães

**Affiliations:** 1 Digital Health and Care, School of Engineering Mathematics and Technology, University of Bristol, Bristol, GBR; 2 Department of Clinical Innovation, Manual, Menwell Ltd, London, GBR; 3 Department of Surgery and Cancer, Imperial College London, London, GBR; 4 Department of Research, Manual, Menwell Ltd, São Paulo, BRA; 5 Department of Research, A Beneficência Portuguesa de São Paulo, São Paulo, BRA

**Keywords:** androgenetic alopecia, finasteride, hair loss treatment, male pattern baldness, norwood classification, oral minoxidil, scalp coverage, service evaluation

## Abstract

Background: Male pattern baldness (MPB) also known as androgenetic alopecia (AA) affects approximately half of men by the fifth decade, and significantly influences both psychological well-being and quality of life. Existing pharmacotherapies include oral finasteride, which reduces dihydrotestosterone (DHT) levels and slows follicular miniaturization, and topical minoxidil, which enhances follicular blood flow and extends the anagen phase. However, adherence to topical regimens can be suboptimal. A combined oral low-dose minoxidil (2.5 mg)-finasteride (1 mg) “All-in-One” regimen could improve convenience, adherence, and possibly efficacy.

Objective: This retrospective study sought to (i) assess the efficacy of a combined oral minoxidil-finasteride therapy over 12 months in a real-world AA cohort; (ii) determine inter-rater reliability of hair loss scoring metrics; and (iii) examine how baseline Norwood severity influences outcomes and effect sizes.

Methods: Data were collected from a UK-based digital health service between January 2020 and December 2023. Eligible men, aged ≥18, with Norwood stages 2-7 AA, initiated combined oral therapy. All had baseline and 12-month follow-up images. The primary outcome was the mean change in hair density according to a clinician-rated 7-point scale. A one-sample t-test compared the mean change to zero (no change). Cohen’s d and Hedge’s g estimated effect sizes, and Cohen’s kappa assessed inter-rater reliability.

Results: Out of 502 men, 92.4% (N = 464) achieved stable or improved outcomes (≥0), and 57.4% (N = 288) showed marked improvements (>0). The inverse-variance weighted mean 7-point change was 0.58 (95% CI: 0.51-0.65; p<0.001; N = 502), indicating significant improvement relative to no change. More severe baseline categories achieved equal or greater gains, with effect sizes up to d≈1.0. Kappa values were modest (Norwood: κ=0.33; 7-point: κ=0.20), indicating fair to slight agreement.

Conclusions: The combined oral minoxidil-finasteride regimen produced statistically significant and clinically meaningful improvements (p<0.001; N = 502) in AA over 12 months. The substantial proportion of stable/improved patients (92.4%, N = 464) and large effect sizes in severe stages highlight its potential. However, modest inter-rater reliability underscores the need for refined protocols or potential AI-driven evaluations. Future prospective trials should confirm these findings and explore optimal dosing, patient selection, and long-term durability.

## Introduction

Background

Male pattern baldness (MPB), also known as androgenetic alopecia (AA), constitutes the most prevalent cause of hair loss in adult men, with incidence increasing significantly with age. By the fifth decade of life, approximately half of all men will exhibit some degree of AA [1]. This condition is typified by a progressive miniaturization of scalp hair follicles, underpinned by genetic predispositions and hormonal factors, most notably dihydrotestosterone (DHT). Although AA poses no direct threat to physical health, its consequences often extend beyond the scalp, exerting a profound influence on psychological well-being, self-esteem, and quality of life [2-4].

For several decades, two pharmacological agents have formed the mainstay of AA therapy: finasteride, an inhibitor of the type II 5α-reductase enzyme that reduces DHT and halts follicular miniaturization [5]; and minoxidil, a vasodilator originally developed as an antihypertensive, later repurposed for its capacity to enhance follicular blood flow, extend the anagen (growth) phase, and promote thicker hair shafts [6,7]. Although finasteride and minoxidil have each shown efficacy in monotherapy (as a once-daily tablet and a typically topical treatment, respectively) adherence remains a key challenge. Topical regimens, in particular, can manifest a plethora of side effects, such as skin irritation, inflammation, and pain, which can be burdensome, and thus, suboptimal in the long term [8].

Recent interest has centered on combining finasteride and minoxidil into a single oral formulation, an “All-in-One” approach. Emerging data on low-dose oral minoxidil have reported promising results, with studies over the past few years highlighting good efficacy and tolerability profiles [9,10]; however, a recent randomized controlled trial (RCT) demonstrated no significant superiority when comparing 5% topical minoxidil with oral formulations [11]. Finasteride remains the cornerstone of AA management [5,7,12]. In principle, the combined oral regimen may afford both convenience and potentially synergistic effects, potentially improving adherence and outcomes. Yet, robust longitudinal data on the efficacy of such dual oral therapy remain limited - existing evidence is primarily based on topical minoxidil in combination with oral finasteride [13,14].

Assessment of AA treatment outcomes depends on reliable and objective measurement tools. The Norwood-Hamilton classification provides a widely used, though static, framework for categorizing hair loss severity. It begins with minimal recession (Type I), progresses through frontotemporal involvement (Types II and III), may include vertex loss (Type III Vertex), and ultimately advances to extensive balding in the higher stages (Types IV-VII), with Type VII representing the greatest extent of hair loss [1,15]. However, the Norwood scale alone offers only a static view of disease extent. More dynamic metrics are required to track therapeutic responses over time. An alternative is a 7-point scale ranging from -3 (substantial deterioration), through 0, (no change), to +3 (marked improvement), however, these tools have their limitations, such as subjectivity in clinical scoring [16-18].

Objectives

Few robust, long-term data exist on combined oral finasteride and low-dose minoxidil, beyond studies focusing on topical minoxidil with oral finasteride. This study addresses that gap by retrospectively analyzing a real-world cohort with moderate to severe AA. Therefore, the present service evaluation study seeks to: (i) assess the efficacy of a combined oral minoxidil-finasteride regimen over a 12-month period in a real-world AA cohort; (ii) examine inter-rater reliability using both the Norwood scale and the 7-point metric; and (iii) explore how baseline severity influences outcomes and effect sizes.

## Materials and methods

Study design and setting

This retrospective service evaluation took place within a UK-based digital health service Menwell Limited (t/a Manual), specializing in the management of hair loss. The data examined were collected between January 2020 and December 2023 as part of routine clinical care. Patients engaged with the service through a secure online electronic health records platform, where their medical histories, consultations, and follow-up assessments were documented. No experimental interventions were introduced beyond standard clinical protocols, and no randomization or prospective recruitment for research purposes was undertaken. All clinical and follow-up data, including images, were collected from routine electronic health records maintained by the digital health service, and extracted retrospectively for this analysis.

Participants

All patients included in this evaluation were male, aged 18 years or older, and had a confirmed clinical diagnosis of AA (commonly within Norwood grades 2-7). Only those who initiated a combined oral minoxidil-finasteride regimen (All-in-one treatment) and maintained adherence for approximately 12 months were included. All patients were entirely naive to pharmacotherapy as “all in one” is a new treatment that was not attainable to the patients prior to enrolling in our service, while others had previously used topical-only regimens and switched to combined oral therapy due to convenience or suboptimal adherence. Ongoing conflicting therapies during the evaluation were excluded. Patients provided standardized and identity anonymized digital scalp images at baseline and at 12-month follow-up. Individuals were excluded if they presented with comorbidities contraindicating oral minoxidil and finasteride (such as uncontrolled cardiovascular conditions), psychiatric histories complicating finasteride use, other forms of alopecia (e.g., areata totalis & universalis, cicatricial alopecia), poor-quality images, or incomplete follow-up data. Patients who underwent concurrent hair restoration surgery or other hair loss interventions during the observation period were also excluded.

Intervention

The intervention under evaluation was the “All-in-One” oral therapy comprising both minoxidil and finasteride in one tablet. Dosages followed the clinic’s standard treatment protocols (Finasteride 1 mg/day, minoxidil at a physician-determined dosage of 2.5 mg/day). Prescriptions were generated by the digital health service and dispensed through the service’s associated pharmacy, which shipped the medication to patients after clinical approval. Patients were counseled at initiation regarding potential benefits, side effects, and the importance of adherence.

Variables

Baseline AA severity was recorded using the Norwood-Hamilton classification, which categorizes AA from minimal recession to advanced patterns of loss (1-7). The primary outcome was the change in hair density and scalp coverage following 12 months of treatment. Change in this hair status was assessed with a 7-point scale ranging from -3 (substantial deterioration) through 0 (no change) to +3 (marked improvement). Both scales were applied to standardized scalp images captured at baseline and at the 12-month follow-up.

Data sources and measurements

All data was sourced from the digital health service’s electronic health records. Each patient self-captured baseline and 12-month scalp photos following standardized instructions regarding camera angle, lighting, and distance, then uploaded the files through the secure platform. To ensure confidentiality, all images were stripped of personal identifiers, as well as block anonymization of the face and assigned coded IDs before review. Clinicians experienced in trichological assessments reviewed these images. The clinicians undertook training for this service evaluation. The training was delivered by a specialist hair loss dermatologist and included examples of the 7-point standardized scoring system used to establish inter-rater reliability. Two independent clinicians evaluated baseline and follow-up images. Discrepancies were resolved by a third clinician who provided an independent assessment. Baseline severity ratings and 12-month changes were documented in the health records. No special calibration sessions were introduced for this evaluation, reflecting typical clinical practice conditions. Clinicians were trained on a sample data set to ensure scoring was an established practice.

As a retrospective evaluation of existing patient records, we were not able to extract information on factors including genetic predispositions or lifestyle factors as they were not routinely recorded in structured data formats during the data collection period. Although the service routinely logs patient-reported side effects through consultation notes, no structured side-effect questionnaire or laboratory data (e.g., liver function tests) was formally collected for this evaluation.

Quantitative variables

The main quantitative variable of interest was the 7-point change in hair status from baseline to 12 months. This variable is ordinal, ranging from -3 to +3. For analytical purposes, summary statistics included the mean and standard deviation of these changes.

Statistical methods and study size

Baseline Norwood scores and 7-point change scores were averaged across the clinical raters. Descriptive statistics (mean, median, standard deviation, minimum, maximum) were calculated for baseline severity and 12-month changes. These baseline severity ratings were retrospectively extracted from the initial patient-submitted images, which is a standard part of routine care and service provision. Inter-rater reliability for both the Norwood classification (at baseline) and the 7-point scale (at follow-up) was assessed using Cohen’s kappa (κ), interpreted according to conventional thresholds. A one-sample t-test against zero determined whether the mean 7-point change differed significantly from no change. Cohen’s d and Hedge’s g were calculated to measure effect sizes. Where category-specific data permitted, an inverse-variance weighted estimate of the overall mean change was considered. Statistical significance was defined as p<0.05, and 95% confidence intervals were reported. Analyses were performed using STATA V18 and Python libraries such as NumPy, Pandas, and SciPy. There was no missing data for the endpoint.

All patients meeting the eligibility requirements and with complete baseline and follow-up data were included. Based on an expected mean improvement of 0.5 points on the 7-point scale (SD ≈ 1.0), a two-sided alpha of 0.05, and a power of 80%, a sample size of approximately 32 participants would be required to detect a statistically significant difference.

Ethical considerations

As a retrospective evaluation of standard clinical care data, this study was determined to be exempt from formal ethical review from a US-based IRB (Institutional Review Board) under 45 CFR §46.104(d)(4) by WIRB-Copernicus Group (WCG) criteria. All patient data was anonymized before analysis to comply with UK data protection laws and the General Data Protection Regulation (GDPR).

## Results

Participant characteristics

**Figure 1:**
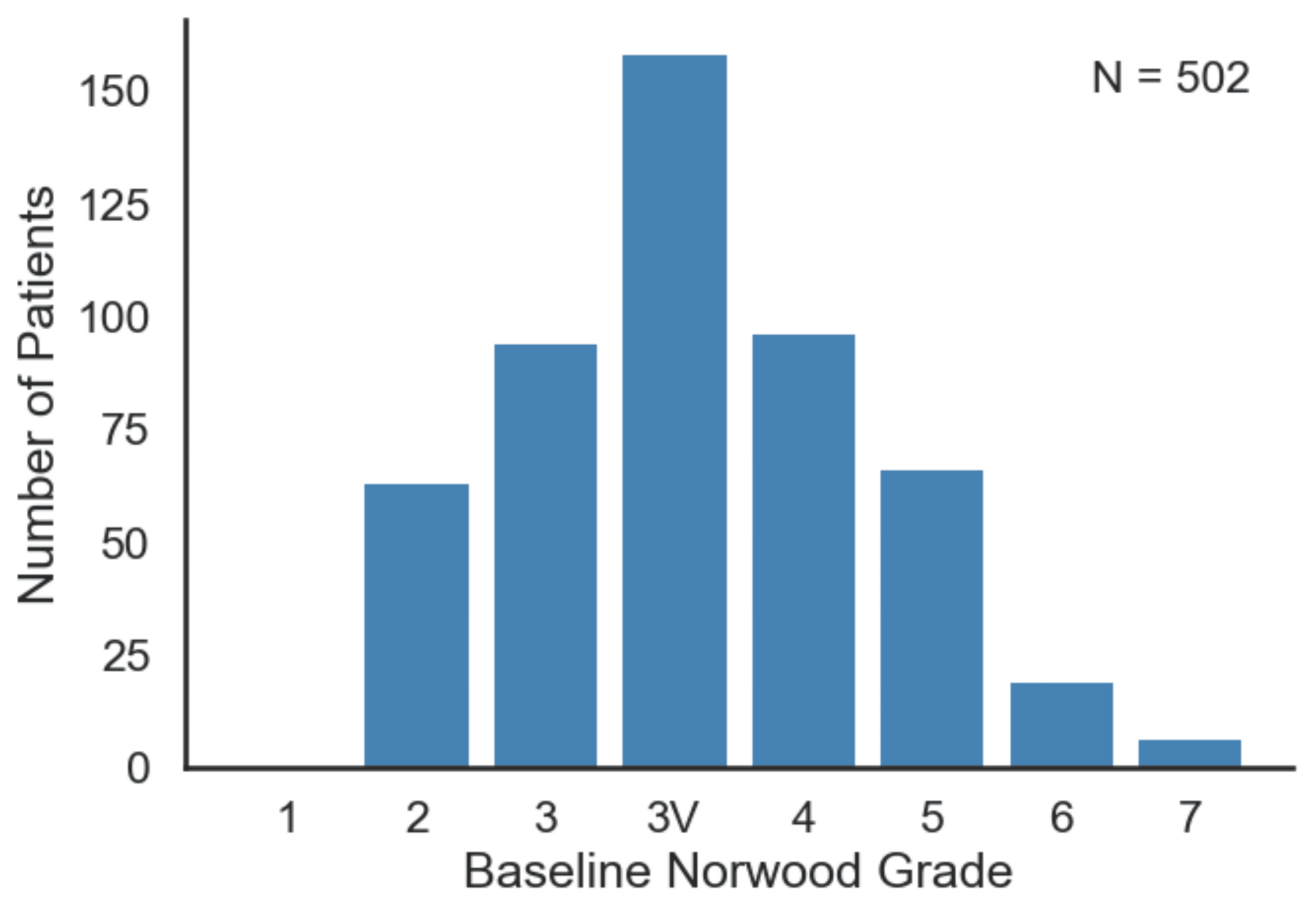
Distribution of patients by average baseline Norwood Grade. A histogram showing the number of patients at each baseline Norwood grade. The Norwood-Hamilton scale categorizes hair loss severity: grade I (minimal or no hairline recession), grade II (triangular frontotemporal recession), grade III (deep temple recession or vertex loss), grade IV (significant frontotemporal and vertex loss separated by a hair band), grade V (larger vertex and frontotemporal loss with a narrowing hair band), grade VI (loss of the hair bridge connecting vertex and frontotemporal regions), and grade VII (severe baldness with a horseshoe-shaped band of sparse hair) [15]. The distribution highlights that most participants presented with moderate patterns of hair loss, such as Norwood grade III or III vertex. The figure was created by the authors of the article.

A total of 540 men initially satisfied the screening criteria; however, 38 were excluded due to poor image quality due to improper lighting and angles, which prevented accurate analysis, leaving 502 men in the final cohort that met the eligibility criteria. Mean age of 35.5 (SD 12.1). Baseline Norwood categories ranged between 2 and 7 as seen in Figure 1, with many individuals presenting moderate patterns of hair loss (e.g., Norwood 3, 3V, or 4).

Primary outcomes: Seven-point changes

**Figure 2:**
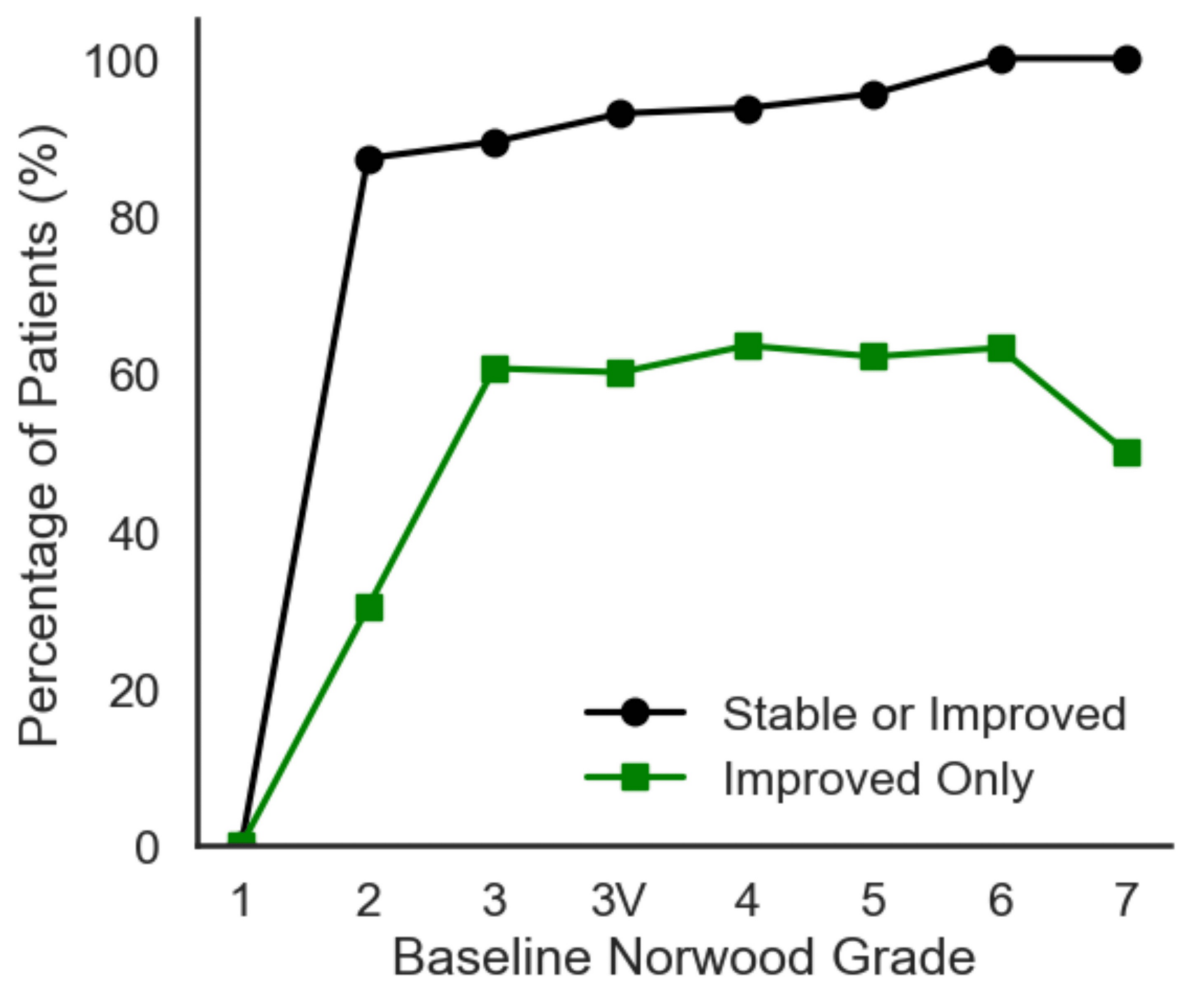
Stable/improved and improved only by baseline Norwood Grade (N = 502). A line chart illustrating the percentage of patients who are stable or improved (≥0) and the percentage who are improved only (>0) across increasing Norwood severities. Although improvement-only rates vary, the majority remain stable or improved in all categories, and advanced stages often display high stable/improved percentages. The figure was created by the authors of the article.

**Figure 3:**
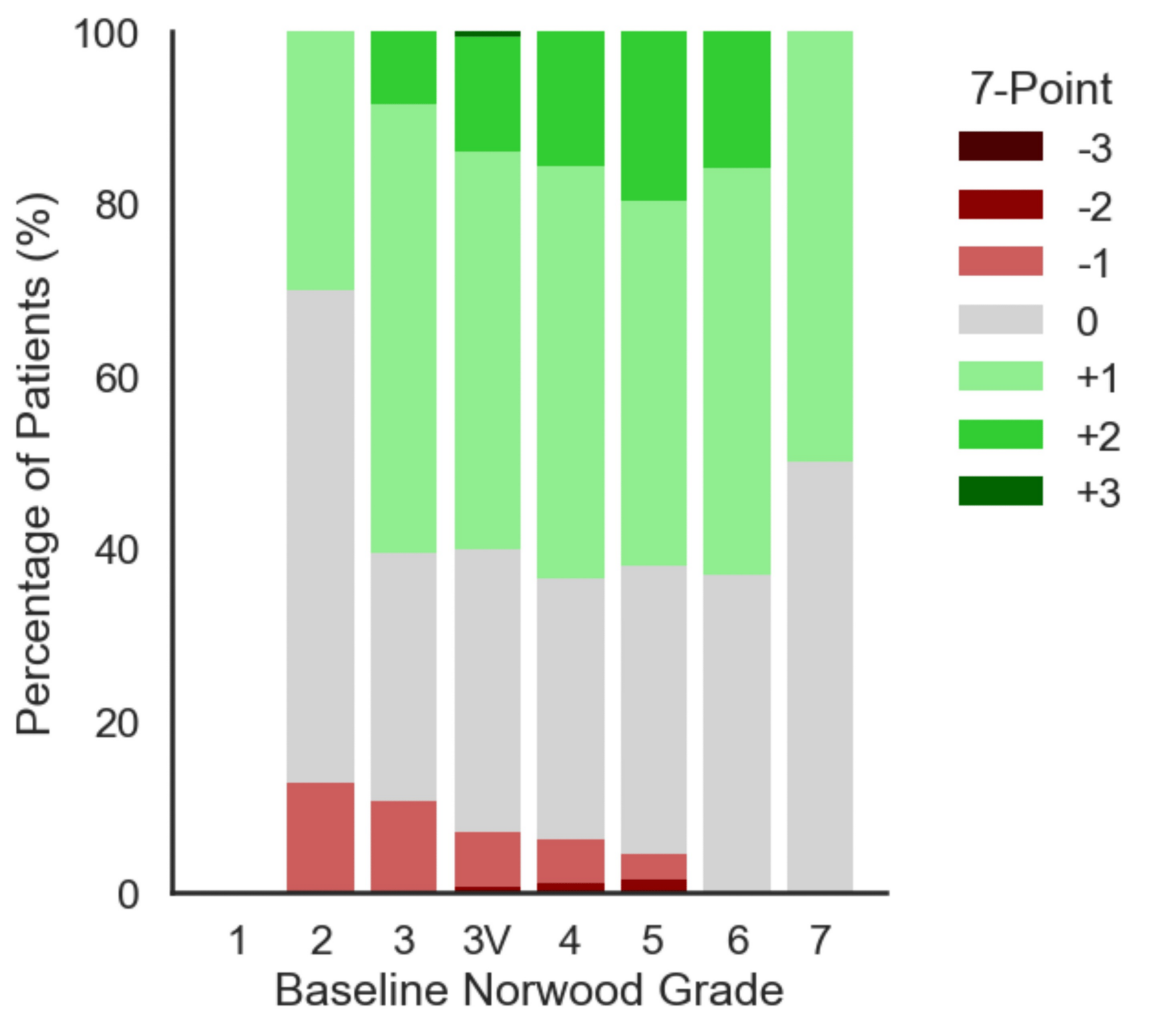
Distribution of 7-point changes by baseline Norwood Grade (N = 502). A stacked bar chart depicting the percentage of patients in each baseline Norwood category distributed across the seven outcome categories (-3 to +3). Green tones represent levels of improvement, gray represents no change, and red tones indicate worsening. The figure shows that even patients with more advanced baseline hair loss frequently achieved stable or improved outcomes. The figure was created by the authors of the article.

A vast majority (92.4%) of patients achieved stable or improved outcomes over the course of 12 months (≥0 on the 7-point scale) as seen in Table 1 and Figure 2. Furthermore, 57.4% exhibited clear improvements (>0)s. Notably, participants with more severe baseline hair loss tended to show equal or greater rates of stability and improvement, culminating in 100% stable or improved outcomes in the most advanced Norwood categories as seen in Table 1 and Figure 3.

**Table 1:** Summary of outcomes by baseline Norwood Category (NW) (N = 502). This table presents the descriptive statistics for baseline Norwood categories, including mean change in 7-point scores, effect sizes, and percentages of stable/improved patients. The statistical test used is a one-sample t-test. One-sample t-tests were used to assess whether the mean change differed significantly from zero. Statistical significance was set at p<0.05.

Baseline NW	N	Mean Change (7-pt)	SD	95% CI (Mean)	p-value	Cohen’s d	Hedges’ g	% Stable/Improved	% Improved
Overall	502	0.616	0.815	(0.544,0.687)	<0.0001	0.76	0.75	92.43	57.37
1 (No data)	–	–	–	–	–	–	–	–	–
2	63	0.175	0.636	(0.014, 0.335)	0.033	0.27	0.27	87.30	30.16
3	94	0.585	0.795	(0.422, 0.748)	<0.0001	0.74	0.73	89.36	60.64
3V	158	0.671	0.833	(0.540, 0.802)	<0.0001	0.81	0.8	93.04	60.13
4	96	0.719	0.829	(0.551, 0.887)	<0.0001	0.87	0.86	93.75	63.54
5	66	0.758	0.860	(0.546, 0.969)	<0.0001	0.88	0.87	95.45	62.12
6	19	0.789	0.713	(0.446, 1.133)	0.0004	1.11	1.06	100	63.16
7	6	0.500	0.548	(-0.075, 1.075)	0.076	0.91	0.77	100	50.00

**Figure 4:**
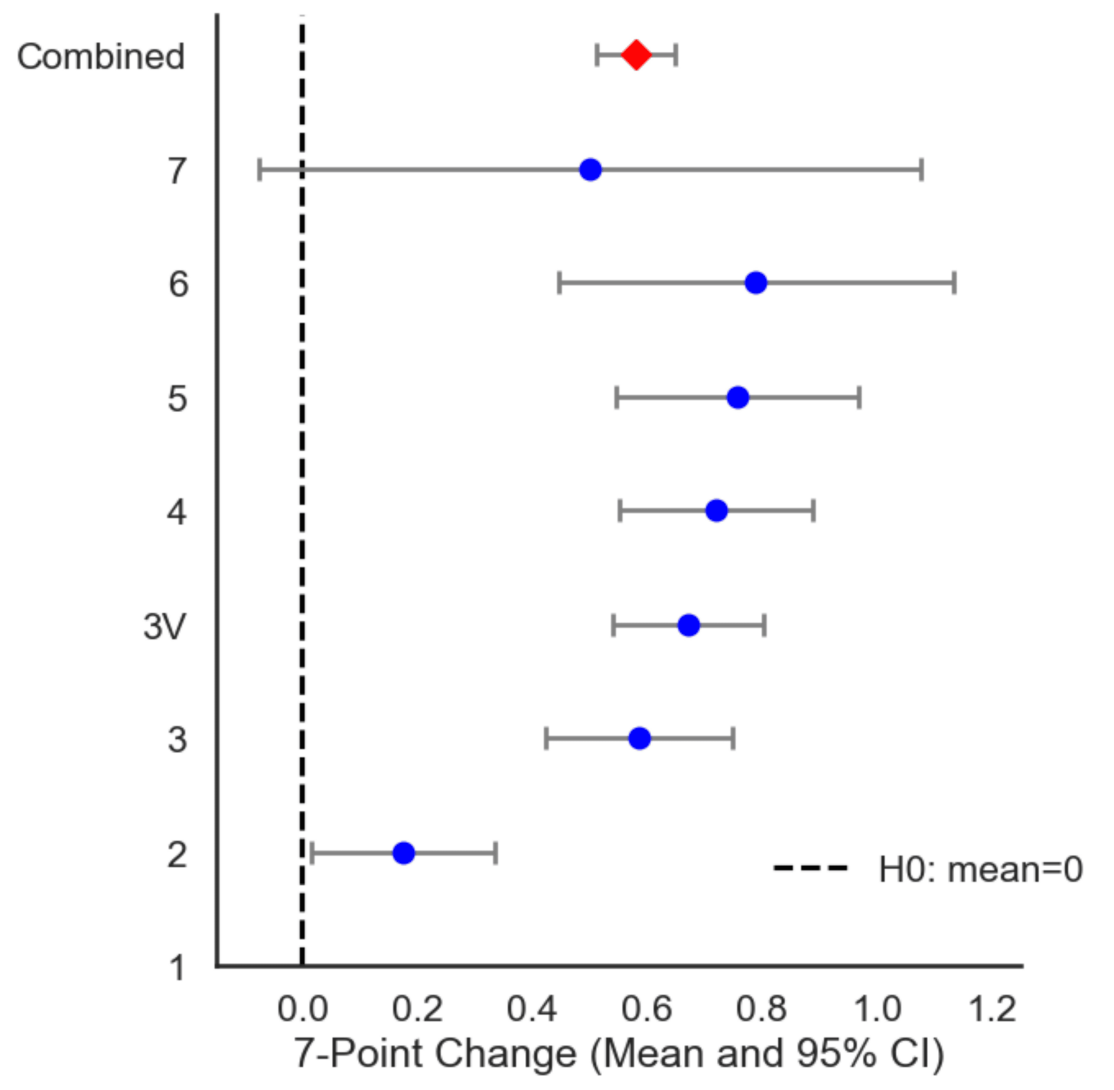
Mean 7-point change by baseline Norwood Category (N = 502). A forest plot displaying the mean 7-point change (with 95% confidence intervals) by baseline Norwood category (y-axis). The vertical dashed line at zero represents no change. Points to the right of zero indicate improvement. The figure suggests that most categories demonstrate positive mean changes and that the magnitude of improvement often increases with baseline severity. The figure was created by the authors of the article.

The 7-point change scores were stratified by baseline Norwood stage - an inverse-variance weighted combined mean 7-point change was calculated as 0.58 (95% CI: 0.51-0.65, N=502, p<0.001). This indicates a highly significant average gain in hair density relative to baseline as seen in Figure 4.

Secondary outcomes and effect sizes

Effect size calculations suggested a correlation between baseline severity and improvement magnitude. Categories with relatively mild initial hair loss displayed smaller effect sizes with a Cohen’s d ≈0.27, indicating modest, yet discernible gains. Whereas, more advanced categories approached or exceeded d=0.7 as seen in Table 1, signifying considerably larger improvements in hair coverage. Such findings emphasize the potential advantage of combined oral therapy even in cases where hair loss is pronounced.

Inter-rater reliability

Kappa values for baseline Norwood classification (0.33) and 7-point changes (0.20) were modest, reflecting fair-to-slight agreement levels as seen in Table 2. Although a third rater harmonized discordant evaluations, these results underscore the subjective element inherent in image-based scoring.

**Table 2:** Inter-rater reliability for Norwood Grades and 7-point changes (N = 2). This table presents the inter-rater reliability assessed between two clinical scorers using Cohen’s kappa for baseline Norwood grades and 7-point changes.

Measure	Kappa	Agreement (%)	Expected Agreement (%)
Norwood at baseline	0.33	46.05	19.77
7-point change	0.2	45.62	31.95

## Discussion

Principal findings

This retrospective evaluation of a combined oral minoxidil-finasteride regimen provides preliminary evidence that such an intervention can yield meaningful improvements in patients with AA, even among those with more advanced baseline severity. However, the overall cohort significantly exceeded the null hypothesis (p<0.001), and the small sample (N=6) in Norwood 7 yields wide confidence intervals crossing near zero. Thus, caution is warranted in definitively rejecting the null for Norwood 7 alone. While longstanding treatments such as oral finasteride and topical minoxidil have individually demonstrated efficacy, adherence remains a key challenge, particularly for topical agents where twice-daily applications may discourage long-term use [9,11,13]. The All-in-One oral formulation examined here potentially addresses some of these limitations by streamlining the therapy, suggesting that ease of administration could improve adherence and thus outcomes.

Comparison with prior work

The observed data indicate that the vast majority of patients (over 92%) maintained or improved their hair density after 12 months, with a substantial proportion (over 57%) exhibiting positive hair growth improvements. Notably, patients with more severe hair loss at baseline not only achieved stabilization but often showed equal or greater gains in hair density. Such findings challenge the traditional assumption that pharmacotherapy is most effective only in relatively mild stages of AA [14,19]. Instead, the results raise the possibility that improved convenience and potential synergistic effects of combined oral therapy may promote clinically relevant benefits even when hair loss is more pronounced.

Both finasteride and minoxidil are well-established monotherapies, with multiple studies and guidelines supporting their utility [5,6,14]. While combined therapy, particularly topical minoxidil plus oral finasteride has shown additive effects [13], evidence for combined oral therapy remains limited [20,21]. Our findings contribute valuable preliminary data, demonstrating clinical efficacy and providing impetus for more comprehensive investigations. The results resonate with recent calls for more patient-centric, data-driven approaches in trichology, potentially altering clinical practice patterns as patients increasingly seek effective, convenient solutions [22-24].

In quantifying these improvements, effect sizes were found to correlate positively with baseline severity, with advanced categories approaching or exceeding Cohen’s d of 1.0. This signals a large treatment effect and offers a sense of clinical importance that goes beyond statistical significance. These data align with emerging literature suggesting that low-dose oral minoxidil combined with finasteride may provide a viable, patient-friendly approach to AA management [9,10], though most prior evidence has focused on topical minoxidil in conjunction with oral finasteride rather than dual oral administration [13,14].

Assessment of treatment outcomes, however, remains complicated by the subjective nature of image-based scoring. The Norwood-Hamilton classification, an established baseline severity tool, and the 7-point scale used to measure change over time both rely on clinician judgment. The modest kappa values (0.33 for Norwood at baseline and 0.20 for 7-point changes) highlight fair-to-slight agreement beyond chance, reflecting persistent variability in inter-rater assessments. Although the involvement of a third adjudicator helped reconcile discrepancies, these findings underscore the need for enhanced standardization. More explicit scoring guidelines, rater training, and standardized image capture protocols could yield greater consistency [14,25]. Moreover, the integration of artificial intelligence (AI) and machine learning-based methods holds promise for reducing subjective bias and ensuring more reproducible assessments of hair density changes, as suggested by recent advancements in trichoscopy and phototrichogram analysis [22].

Limitations

Several limitations must be considered. First, the retrospective design inherently restricts the ability to control confounders and confirm causality. Selection bias may be present, as only those who completed the 12-month follow-up and provided adequately usable images were included, 38 individuals were specifically excluded due to poor image quality. This approach can skew outcomes toward a more motivated or adherent cohort. Confounding factors such as patient age, genetic predispositions, lifestyle, and varying adherence rates were not explicitly accounted for. Although excluding patients with contraindications or unsuitable scalp conditions helped refine the cohort, it may have reduced generalizability. Additionally, the absence of a control or placebo group, along with no randomization or blinding, precludes definitive causal inference that observed improvements resulted solely from the combined oral therapy. The one-sample t-test comparing mean 7-point changes to zero assumes that no change is a suitable null hypothesis, which might not fully capture the complexity of AA progression. Larger, prospective, randomized controlled trials would be required to provide stronger evidence of efficacy and safety, while also allowing the incorporation of patient-reported outcomes to gauge quality of life improvements, adherence tracking, and long-term cost-effectiveness.

Regarding side-effects monitoring, we did not systematically collect laboratory data (e.g., liver function tests) or structured side-effect questionnaires, which precludes a thorough assessment of potential systemic effects over 12 months. While the service offers patient consultations for reported side effects, no comprehensive adverse-event dataset was available at the time of analysis. As a result, any unreported or unrecorded effects are not reflected here, and further prospective or dedicated pharmacovigilance studies are recommended.

Moreover, the evaluation relied on clinician-judged outcomes derived from standardized images rather than objective quantitative measures such as hair counts, phototrichogram data, or trichoscopy-based follicular analyses [26,27]. The absence of these more objective and precise methods may have limited the accuracy and resolution of outcome assessments. Incorporating hair counts, shaft diameter measurements, and trichoscopy endpoints in future research could provide more granular and reproducible data, mitigating subjectivity and variability. Finally, we are aware that Cohen’s d can be less illustrative for ordinal scales and does not fully capture all aspects of clinical significance, however, we utilized mean changes in hair density with established p-values for significance.

Future directions

Further retrospective analysis of Menwell Limited’s datasets could provide helpful insights. Exploring medication adherence, side-effect rates, and the natural progression of treatment with combination therapies could enhance the context of our findings. Additional investigations should also be conducted on emerging therapies for AA such as dutasteride, a more potent DHT-blocker [20,28].

Prospective, randomized controlled trials comparing combined oral therapy against monotherapies, topical regimens, or other emerging interventions are warranted to confirm these preliminary insights and establish definitive efficacy and safety profiles. Incorporating patient-reported outcome measures (PROMs) [29,30] would help capture improvements in quality of life and psychological well-being, extending the clinical relevance of the data. Long-term follow-up beyond 12 months could elucidate durability, maintenance strategies, and cost-effectiveness. Moreover, leveraging AI-driven methods to objectively quantify hair parameters such as follicle counts, shaft diameters, and growth rates may address current limitations in subjective assessments and enhance the precision of future studies.

## Conclusions

A vast majority (92.4%) of patients (N = 464) saw their AA halted or improve over 12 months. This evaluation indicates that a combined oral minoxidil-finasteride regimen benefits even the most severe cases of AA. The findings suggest improved convenience and potential synergy, while modest inter-rater reliability highlights the need for enhanced assessment methodologies. Future retrospective data analyses and prospective trials that incorporate PROMs and AI-assisted analytics will contribute to this nascent knowledge base relating to the clinical management of AA.
